# Transcriptome profiling across 6 different tissues in *Vigna radiata* var. *sublobata*

**DOI:** 10.1186/s12863-025-01403-5

**Published:** 2025-12-20

**Authors:** Zi-Meng Sun, Xue Chen, Qi-Chao Wu, Song Hou, Zhi-Wei Wang, Min Liu, Guan Li, Kai-Hua Jia, Peng-Fei Chu, Na-Na Li

**Affiliations:** 1 College of Agriculture and Biology, Liaocheng University, Liaocheng, Shandong 252000 China; 2 National Saline-Alkali Tolerant Crop Germplasm Resources Nursery (Dongying), Jinan, Shandong 250100 China; 3 Shandong International Joint Laboratory of Agricultural Germplasm Resources Innovation, Jinan, Shandong 250100 China; 4 Institute of Crop Germplasm Resources, Shandong Academy of Agricultural Sciences, Jinan, Shandong 250100 China; 5 Legume Crops Research Institute, Weifang Academy of Agricultural Sciences, Weifang, Shandong 261017 China; 6 College of Forestry, Key Laboratory of State Forestry Administration for Silviculture of the Lower Yellow River, Shandong Agricultural University, Taian, Shandong 271017 China

**Keywords:** *V. radiata* var. *sublobata*, Transcriptomics, Differential expression analysis, Wild fabaceae plant resources

## Abstract

**Objective:**

Mung bean (*Vigna radiata*) is a leguminous crop of significant economic, nutritional, and ecological importance. Its wild relative, *V. radiata* var. *sublobata*, is a wild legume exhibiting strong environmental adaptability and potential economic value. Its rich genetic diversity serves as a critical resource for research on crop stress resistance, thereby offering valuable wild genetic material for mung bean varietal improvement. In this study, we generated transcriptome sequencing data from six distinct tissues of *V. radiata* var. *sublobata*, providing a fundamental data for functional genomics research and molecular breeding applications.

**Data description:**

In this study, we performed systematic transcriptomic sequencing analysis on six different tissues of *V. radiata* var. *sublobata*, with three biological replicates for each tissue type. Principal component analysis (PCA) demonstrated a significant separation trend among different tissue samples. A total of 19,405 differentially expressed genes (DEGs) were identified through pairwise cross-tissue comparisons. Gene Ontology (GO) enrichment analysis revealed that, relative to leaf tissue, seed tissue exhibited significant up-regulation of genes implicated in energy metabolism, storage processes, and developmental regulation. This study not only provides a tissue-specific gene expression profile for *V. radiata* var. *sublobata* but also offers important theoretical foundations and genetic resources for the utilization of wild legume plant resources and molecular breeding.

## Objective

Mung bean (*Vigna radiata*) is a nutritionally rich and environmentally sustainable crop [1, 2]. It is an excellent source of high-quality protein and essential nutrients, serving both as a health-promoting dietary component and as a nitrogen-fixing agent that enhances agricultural sustainability [1, 3, 4]. Due to its short growth cycle and strong adaptability, mung bean plays a profound role in agricultural sustainability and holds significant scientific value in legume genetic research [5].

*V. radiata* var. *sublobata*, considered the wild ancestor of mung bean, is primarily distributed in eastern, southern, and southwestern regions of China. It exhibits high drought tolerance, adaptability to poor soils, and possesses medicinal, edible, and ecological value [6, 7]. Previous studies have primarily focused on bruchid resistance, salt tolerance, and hybrid compatibility [8–10]. These studies provide a scientific foundation for its rational utilization and underscore its significance as a promising novel food resource globally [11]. Nevertheless, the underlying molecular mechanisms governing gene expression remain largely unexplored.

Here, leveraging our previously assembled Telomere-to-Telomere (T2T) reference genome of *V. radiata* as the reference [12], we successfully delineated a cohort of differentially expressed genes (DEGs) exhibiting pronounced expression reprogramming via comparative transcriptome profiling across multiple tissues. These DEGs provide crucial insights into the unique biological mechanisms of *V. radiata* var. *sublobata*.

## Data description

### Sample collection

The seeds of *V. radiata* var. *sublobata* were collected from the experimental field of the Weifang Academy of Agricultural Sciences (Weifang, Shandong Province, China). These wild accessions were taxonomically identified as *V. radiata* var. *sublobata* by Dr. Qichao Wu, a plant taxonomy specialist at Shandong Agricultural University. Moreover, sequence analysis indicated that these materials share a high genomic similarity with *V. radiata*, with an identity exceeding 99% (Liu et al., 2026, unpublished data). The seeds were germinated indoors by breaking the skin with a small knife and soaking them in water for three days. During the flowering period, uniformly growing plants were selected. Sterilized scissors were used to collect roots (secondary root system approximately 5 cm from the root tip), stems (3rd–4th internodes), mature leaves (the 3rd fully expanded leaf from the top), flowers (fully open corolla). At 10 days post-flowering, additional samples were collected young seeds, and pods (young pods 5–8 cm in length). All samples were immediately flash-frozen in liquid nitrogen for 10 min, transferred to a -80 °C ultra-low temperature freezer for storage, and kept until RNA extraction. To minimize the influence of circadian rhythms, sampling was uniformly conducted between 9:00 and 11:00 AM.

### RNA extraction, library construction, and transcriptome sequencing

Total RNA was extracted from *V. radiata* var. *sublobata* tissue using an RNA extraction kit (Biospin, Hangzhou). Briefly, powdered plant tissue was lysed in AG buffer supplemented with 2% β-mercaptoethanol and homogenized by shaking. After centrifugation, transfer the supernatant to a new centrifuge tube, add an equal volume of anhydrous ethanol, mix thoroughly, transfer to a spin column, and centrifuge to remove the liquid from the collection tube. Add wash buffer for washing and centrifuge to remove the liquid. Prepare the DNase I working solution, add it to the spin column, let it stand, and then centrifuge. Continue to add PG buffer and wash buffer to wash, removing any residual ethanol. Finally, add the RElution buffer and centrifuge to obtain total RNA.

Use Oligo (dT) magnetic beads to specifically enrich eukaryotic polyadenylated mRNA from total RNA, then randomly fragment the mRNA into suitable fragments under optimized fragmentation buffer conditions. Using the fragmented products as templates, synthesize the first strand of cDNA using random hexameric primers and M-MLV reverse transcriptase, followed by DNA polymerase I and RNase H to complete double-stranded cDNA synthesis. Perform end repair on the double-stranded cDNA and add a 3’-A overhang, then ligate DNBSEQ-compatible sequencing adapters with sample-specific indices. Target fragments were selected using AMPure XP magnetic beads and amplified through 12 cycles of PCR to construct the final cDNA library. Strict quality control was performed using an Agilent 2100 Bioanalyzer to ensure that the library fragment distribution was concentrated within the 300 ± 50 bp range and free of adapter dimer contamination [13]. High-quality libraries were sequenced on the DNBSEQ-T7 platform, generating 150 bp paired-end sequences.

### Gene expression analysis

Data preprocessing was performed using fastp v0.12.4 [14] to remove low-quality reads (Q < 20) and adapter sequences, resulting in clean reads with an average retention rate of 94.5%–96.2% [20] (Data file 1). Subsequently, the clean reads were aligned to the T2T reference genome of *V. radiata* [12] using salmon v1.6.0 [15] with the --validateMappings and --numBootstraps 100 parameters. The average mapping percentage (proportion of clean reads aligned to the reference genome) across all libraries was 81.53% [20] (Data file 1). Transcript expression levels (Transcripts Per Million, TPM) were then calculated based on the aligned reads. Subsequently, tximport v1.22.0 was used to aggregate transcript-level expression data to the gene level [16]. After filtering out 4,624 non-expressed genes (TPM < 1 in all samples), principal component analysis (PCA) was performed on the TPM values (Data file 2). Differential expression analysis was performed using DESeq2 v1.4.5 [17], Differentially expressed genes (DEGs) were identified the following criteria: an adjusted *p*-value (Benjamini–Hochberg correction for multiple testing) ≤ 0.05 and an absolute log_2_ (fold change) ≥ 1. Functional annotation of *V. radiata* var. *sublobata* protein sequences was performed using eggNOG-mapper v2 [18]. The clusterProfile package was employed to perform Gene Ontology (GO) pathway enrichment analyses on differentially expressed genes (DEGs) [19].

PCA based on TPM values demonstrated clear clustering among different tissues (Date file 2) [20]. Compared seeds, the upregulated genes in *V. radiata* var. *sublobata* pods were significantly enriched in processes such as photosynthesis, light reactions, and isoprenoid biosynthesis, indicating that the pods possess photosynthetic capacity (Date file 3) [20]. Similarly, comparisons between the roots and leaves of *V. radiata* var. *sublobata* revealed that genes associated with energy metabolism and storage, as well as developmental regulation, were highly expressed in seeds, further supporting this possibility (Date file 3) [20].

**Table:** 

Label	Name of data file/data set	File types (file extension)	Data repository and identifier (DOI or accession number)
Data file 1	Raw data statistics	Microsoft Excel spreadsheet (.xlsx)	Zi-Meng Sun. Transcriptome analysis and validation data support. 2025. Figshare (10.6084/m9.figshare.30084517.v5) [20]
Data file 2	Principal component analysis	Portable document format (.pdf)	Zi-Meng Sun. Transcriptome analysis and validation data support. 2025. Figshare (10.6084/m9.figshare.30084517.v5) [20]
Data file 3	GO analysis	ZIP file (.zip)	Zi-Meng Sun. Transcriptome analysis and validation data support. 2025. Figshare (10.6084/m9.figshare.30084517.v5) [20]
Data file 4	readme	Temporary Edit Text (tet)	Zi-Meng Sun. Transcriptome analysis and validation data support. 2025. Figshare (10.6084/m9.figshare.30084517.v5) [20]
Data set 1	Transcriptome sequencing in *V. radiata* var. *sublobata*	Fastq file (fastq.gz)	GSA (https://ngdc.cncb.ac.cn/gsa/browse/CRA024186) [21]

## Limitation

This study is based on transcriptomic sequencing analysis of six representative tissues, providing valuable insights into gene expression characteristics. However, sampling was limited to several conventional tissues without fine-scale dissection — for example, anther and petal tissues were not specifically sampled. Moreover, sampling was conducted at only two developmental stages, without covering the entire developmental process.

All experiments were performed under normal laboratory conditions without any stress treatments, and thus stress-related expression profiles were not included. In addition, the study focused solely on transcript-level analyses, while post-transcriptional regulatory mechanisms such as miRNA-mediated regulation and DNA methylation were not explored.

## Data Availability

The data that support the findings of this study have been deposited into Genome Sequence Archive (GSA) of China National Center for Bioinformation (CNCB) with accession number CRA024186.

## Abbreviations

PCA: Principal Component Analysis
GO: Gene Ontology
DEG: Differential Expressed Gene
T2T: Telomere-to-Telomere
TPM: Transcripts Per Million
